# Attenuation of Live-Attenuated Yellow Fever 17D Vaccine Virus Is Localized to a High-Fidelity Replication Complex

**DOI:** 10.1128/mBio.02294-19

**Published:** 2019-10-22

**Authors:** Emily H. Davis, Andrew S. Beck, Ashley E. Strother, Jill K. Thompson, Steven G. Widen, Stephen Higgs, Thomas G. Wood, Alan D. T. Barrett

**Affiliations:** a Department of Pathology, The University of Texas Medical Branch, Galveston, Texas, USA; b Department of Biochemistry and Molecular Biology, The University of Texas Medical Branch, Galveston, Texas, USA; c Department of Microbiology & Immunology, The University of Texas Medical Branch, Galveston, Texas, USA; d Sealy Institute for Vaccine Sciences, The University of Texas Medical Branch, Galveston, Texas, USA; e Biosecurity Research Institute, Kansas State University, Manhattan, Kansas, USA; Baylor College of Medicine

**Keywords:** yellow fever, attenuation, vaccine, ribavirin, quasispecies, live virus vaccine

## Abstract

The molecular basis of attenuation for live-attenuated vaccines is poorly understood. The yellow fever (YF) 17D vaccine virus was derived from the wild-type, parental strain Asibi virus by serial passage in chicken tissue and has proven to be a very safe and efficacious vaccine. We have previously shown that wild-type Asibi is a typical RNA virus with high genetic diversity, while the 17D vaccine virus has very little genetic diversity. To investigate this further, we treated Asibi and 17D viruses with ribavirin, a GTP analog with strong antiviral activity that increases levels of mutations in the viral genome. As expected, ribavirin treatment introduced mutations into the Asibi virus genome at a very high frequency and decreased viral infectivity while, in contrast, the 17D vaccine virus was resistant to ribavirin, as treatment with the antiviral introduced very few mutations into the genome, and viral infectivity was not lost. The results were confirmed for another YF wild-type parental and vaccine pair, a wild-type French viscerotropic virus and French neurotropic vaccine. Using recombinant Asibi and 17D viruses, ribavirin sensitivity was located to viral nonstructural genes. Thus, two live-attenuated YF vaccine viruses are genetically stable even under intense mutagenic pressure, suggesting that attenuation of live-attenuated YF vaccines is due, at least in part, to fidelity of the replication complex resulting in high genetic stability.

## Abstract (precis)

Live-attenuated viral vaccines are highly safe and efficacious but represent complex and often multigenic attenuation mechanisms. Most of these vaccines have been generated empirically by serial passaging of a wild-type (WT) virus in cell culture. One of the safest and most effective live-attenuated vaccines is yellow fever (YF) virus strain 17D, which has been used for over 80 years to control YF disease. The availability of the WT parental strain of 17D, Asibi virus, and large quantities of clinical data showing the effectiveness of the 17D vaccine make this WT parent/vaccine pair an excellent model for investigating RNA virus attenuation. Here, we investigate a mechanism of 17D attenuation and show that the vaccine virus is resistant to the antiviral compound ribavirin. The findings suggest that attenuation is in part due to a low probability of reversion or mutation of the vaccine virus genome to WT, thus maintaining a stable genotype despite external pressures.

## INTRODUCTION

The genus Flavivirus contains approximately 70 viruses, most of which are arthropod-borne (arboviruses) and major public health problems, including dengue, Japanese encephalitis, yellow fever (YF), and Zika viruses. These enveloped, positive-sense RNA viruses contain the following 10 genes: three structural proteins (capsid [C], membrane [M], and envelope [E]) and seven nonstructural (NS) proteins (NS1, NS2A, NS2B, NS3, NS4A, NS4B, and NS5). The functions of the NS proteins, which constitute the host cell membrane-bound replication complex, are incompletely understood. The viral protease and helicase are contained within NS3, and methyltransferase and polymerase reside in NS5 (1, 2). Yellow fever virus (YFV) is the prototype member of the genus and the etiological agent of yellow fever, so named for the jaundice caused by advanced infection of the liver. YFV is endemic to tropical South America and sub-Saharan Africa, where it causes periodic, seasonal outbreaks of YF. As there are no approved antiviral therapies for YFV, the disease is controlled primarily by a very successful live-attenuated vaccine (LAV) virus, strain 17D (3).

It is well known that the adaptation of RNA viruses to cell or tissue culture by serial passage alters virus tropism. Such empirical derivation methods have been used to generate many of the currently utilized viral LAVs, including LAVs used to prevent YF, rubella, polio, mumps, and measles. The 17D vaccine virus was derived in the 1930s by 176 serial passages of wild-type (WT) strain Asibi virus in various cell types, including chicken embryos lacking nervous tissue, wherein it lost viscerotropic properties as well as the ability to be transmitted by mosquitoes (4). Although the amino acid substitutions that distinguish 17D from its parent, WT virus Asibi, have been reported and constitute an internationally standardized vaccine genotype, the mechanism(s) of attenuation of the 17D vaccine are poorly understood and are likely to originate from the combined contribution of structural and NS genes (5). A recent comparison of WT Asibi virus and 17D vaccine virus by next-generation sequencing (NGS) revealed that Asibi virus was genetically heterogeneous, which is typical for an RNA virus, while the 17D-204 substrain vaccine virus contained limited intra- and interpopulation variability. Subsequent vaccine lot stability studies confirm this lack of diversity, which has been proposed to contribute to the attenuation and excellent safety record of the 17D vaccine (6).

Concurrent to the development of the 17D vaccine virus, another live-attenuated YF vaccine, French neurotropic vaccine (FNV) virus, was developed by 128 passages of the WT strain French viscerotropic virus (FVV) in the mouse brain. Asibi virus and FVV were isolated in the same YF outbreak in West Africa in 1927. As such, these viruses are genetically very similar, with only two amino acid mutations differentiating the WT strains (E-200 and NS3-280) (7). Although the 17D and FNV virus vaccine strains were both developed similarly through empirical serial passage, the vaccines share only two common substitutions at M-36 and NS4B-95. FNV virus was used during mass vaccination campaigns in Francophone Africa until 1984, when it was taken off the market following observation of postvaccinal encephalitic events in children. Like 17D, FNV virus was recently shown to have reduced levels of diversity compared to FVV, suggesting that live-attenuated YFV vaccine viruses may share common mechanisms of attenuation (7).

The genomes of RNA viruses consist of a heterogeneous population of RNA species, termed quasispecies or mutant spectrum, that differ in nucleotide sequence and constitute an aggregate unit of selection. This variability is derived from error-prone viral RNA-dependent RNA polymerases (RdRps) and a high mutation rate with considerable nucleotide mismatching (8). Though the majority of mutations produced by a high mutation rate are deleterious, the production of a spectrum of genotypes likely confers a fitness advantage in surmounting population bottlenecks (9). This is extremely beneficial to arboviruses that move between diverse hosts in their transmission cycles. High mutation rates are favored in these changing environments, as diverse populations are more likely to contain random mutations that may be helpful in the new environment (10–12). This was shown using St. Louis encephalitis virus, where a decrease in genetic diversity correlated to decreased infectivity in chickens and mosquitoes. Interestingly, in this study, the method of genetic restriction was adaptation to cell culture (13). Conversely, during serial passage, viruses adapt to an unchanging cell culture system. Without the selection pressure of a changing environment, the virus may instead produce a consensus sequence that is highly fit and highly stable, with random mutation no longer providing a fitness advantage (14). Though the RdRp is the most obvious contributor to changes in the mutation rate, it has been shown (including with YFV) that genes outside the RdRp also influence viral diversity (15–17).

RNA virus diversity in attenuated mutants has been investigated. Studies with poliovirus have shown that increasing the fidelity of the RdRp attenuates the virus by reducing the ability of the virus to enter the brain and cause disease (8, 18). When the high-fidelity poliovirus mutant was supplemented with WT virus, both viruses were able to enter and replicate in the brain. Similarly, a high-fidelity variant of chikungunya virus was used to illustrate the importance of a heterogeneous mutant spectrum in arbovirus fitness as the virus moved between mammalian and mosquito hosts (19). High-fidelity replication complexes, and the fitness decrease they confer, are attractive qualities for live-attenuated vaccines, as these viruses are presumably less dynamic than WT virus in the host and present reduced risk of reversion to pathogenicity. While studies with attenuated mutants are valuable, there has been very little published on the genetic diversity of licensed live-attenuated vaccines (7, 20–24).

Lethal mutagenesis is an antiviral principle that utilizes the concept of error threshold, a theoretical maximum rate of genetic diversity. Antivirals utilizing this mechanism of action are typically nucleoside analogs which, when incorporated into the genome, increase the viral mutation rate beyond the error threshold. At this point, the benefits of a robust mutant spectrum are outweighed by the accumulation of deleterious mutations and the generation of defective genomes that inhibit viral replication and reduce infectivity (25). Ribavirin, a GTP nucleoside analog, has been shown to be effective against a large number of RNA viruses, including Lassa and hepatitis C viruses (26–28). Like other nucleoside analogs, ribavirin is believed to increase the mutation rate of RNA viruses upon incorporation, leading to lethal mutagenesis (29, 30). High-fidelity RdRps do not recognize or incorporate the drug, which leads to a resistance phenotype, and as such, high-fidelity viruses can be selected, such as the poliovirus mentioned above (8). Interestingly, the paradigm for YFV is that it is resistant to ribavirin, so it has not been investigated as an effective antiviral for YFV infection (31) This conclusion was based on studies with 17D vaccine virus where ribavirin was only effective against the virus at cytotoxic concentrations. The mechanism cited in these studies was not lethal mutagenesis but instead inhibition of IMP dehydrogenase (IMPDH) by ribavirin, leading to a reduction in intracellular GTP pools. The WT Asibi strain was never tested (31).

Due to the differences in genetic diversity between WT Asibi virus and its 17D vaccine virus derivative, we investigated the changes in genetic diversity of 17D and Asibi viruses following treatment with ribavirin. It was found that 17D virus was resistant to antiviral effects, whereas the drug was active against WT Asibi. Furthermore, ribavirin resistance was observed in a second live-attenuated YFV vaccine strain (FNV) and susceptibility in a second WT YFV strain (FVV). Experiments with recombinant-derived mutant viruses indicated that resistance was associated with the NS genes of the vaccine virus, suggesting a role for the replication complex in susceptibility. The contribution of anti-IMPDH activity was tested using mycophenolic acid (MPA), a nonnucleoside triphosphate antiviral that inhibits IMPDH and has been shown to be effective against 17D (31, 32). WT and vaccine strains of YFV were of equivalent sensitivity to MPA *in vitro*, supporting the hypothesis that the incorporation of a GTP analog is the primary mechanism of action of ribavirin against WT YFV. We propose that YFV vaccine strains are resistant to ribavirin due to an acquired high-fidelity replication mechanism and that this contributes to the attenuated phenotype.

## RESULTS

### Nucleoside analogs show greater *in vitro* antiviral activity against WT YFV than against vaccine YFV strains.

**FIG 1:**
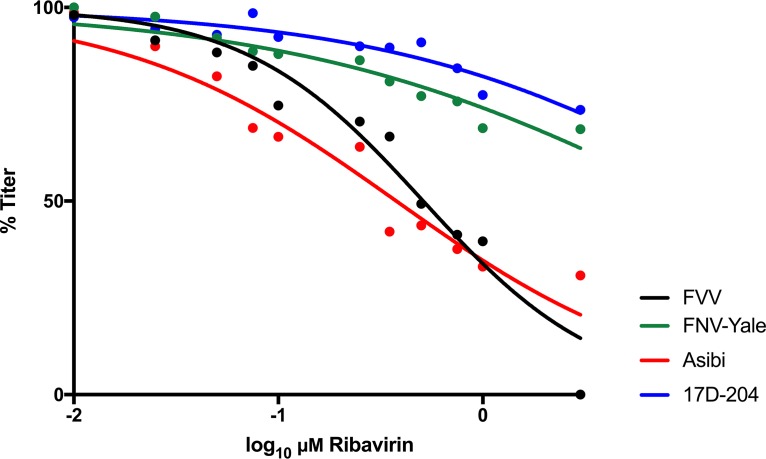
Dose response of YFV vaccine and WT strains to *in vitro* ribavirin treatment. 17D-204, Asibi, FNV, and FVV were incubated with the GTP nucleoside analog ribavirin in Vero cells. After 48 h, the supernatant was collected and titrated for viral load (focus-forming units [FFU]). The titers at each concentration were normalized to untreated, infected cells and fit using a dose-response nonlinear regression. The experiment was undertaken in triplicate, and the points shown are an average of the results from these experiments.

The effect of ribavirin treatment on the viral infectivity of low-passage-number WT Asibi virus and commercial 17D vaccine virus strain was investigated in Vero cells. The viral infectivity reduction curves for WT Asibi virus and vaccine YFV were significantly different (*P < *0.0001). The 50% inhibitory concentration (IC_50_) values of Asibi (0.37 μM, *R*^2^ = 0.94) and 17D (21.17 μM, *R*^2^ = 0.88) viruses differed by 57-fold, showing that ribavirin is much more active against Asibi virus than against 17D virus (Fig. 1). The multiplication kinetics of Asibi and 17D viruses are indistinguishable in Vero cells, which suggests that the neither virus has a replicative advantage at incorporating the drug (data not shown). Furthermore, the studies were undertaken using the same multiplicity of infection (MOI) of virus (0.05) to control for the stock titer. In order to determine if the trends observed between 17D and Asibi viruses were strain specific or otherwise consistent with the attenuation of YFV, the studies were repeated with the WT FVV and its live-attenuated vaccine derivative FNV virus. The FVV and FNV virus curves were significantly different from each other (*P < *0.037). The IC_50_ values of FVV (0.51 μM, *R*^2^ = 0.93) and FNV (10.59 μM, *R*^2^ = 0.72) viruses differed by 14-fold showing that, again, WT YFV was more susceptible to ribavirin than was a live-attenuated vaccine virus. It was noted that YFV vaccine strains 17D and FNV were not statistically different from each other (*P > *0.999). The same was also true in a comparison of the two WT YFV strains, where no difference can be detected between the curves (*P > *0.999).

### YFV strains are equally susceptible to MPA, an antiviral without a lethal mutagenesis mechanism.

**FIG 2:**
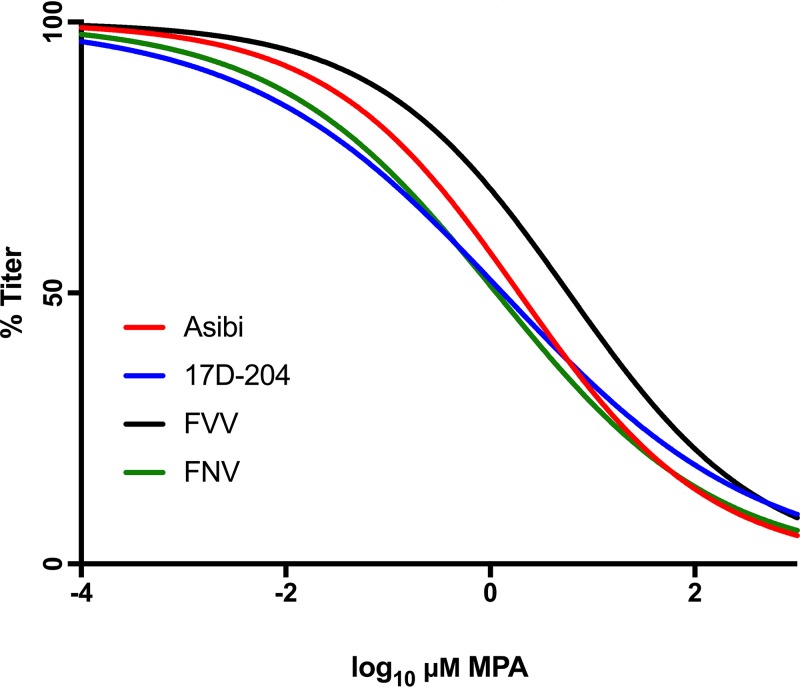
Dose response of 17D-204 vaccine, Asibi, FNV, and FVV to *in vitro* MPA treatment. 17D-204, Asibi, FNV, and FVV were incubated with the IMPDH inhibitor MPA in Vero cells. After 48 h, the supernatant was collected and titrated for viral load (FFU). The titers at each concentration were normalized to untreated, infected cells and fit using a dose-response linear regression. The experiment was undertaken in triplicate, and the points shown are an average of the results from these experiments.

Ribavirin is known to have two mechanisms of action. The first involves incorporation of the drug, a guanine nucleoside analog, into the genome that increases mutation frequency leading to error catastrophe for the virus, and the second is inhibition of IMDPH that decreases the pool of GTP and increases the incorporation of ribavirin. To investigate the alternate mechanism of action of ribavirin activity, the effect of antiviral MPA treatment on the viral infectivity of the WT (Asibi virus and FVV) and vaccine strains (17D and FNV viruses) was investigated. MPA is an anti-IMPDH antiviral with no nucleoside triphosphate (NTP) analog activity. Viral infectivity reduction curves of MPA-treated Asibi and 17D viruses were not significantly different (*P* = 0.68), and the IC_50_ values of Asibi (1.91 μM, *R*^2^ = 0.96) and 17D (1.32 μM, *R*^2^ = 0.93) viruses were statistically indistinguishable and within the range considered safe and effective for MPA *in vitro* drug screens (33) (Fig. 2). The same was true for FVV (5.82 μM, *R*^2^ = 0.94) and FNV (1.15 μM, *R*^2^ = 0.83) viruses, with reduction curves and IC_50_ values which appeared to be statistically similar (*P > *0.999) (Fig. 2). These findings suggest that drugs that inhibit IMPDH exclusively are universally effective against YFV, while drugs that increase the error rate of RNA viruses are only effective against WT YFV strains.

### Effect of ribavirin on genetic diversity.

**FIG 3:**
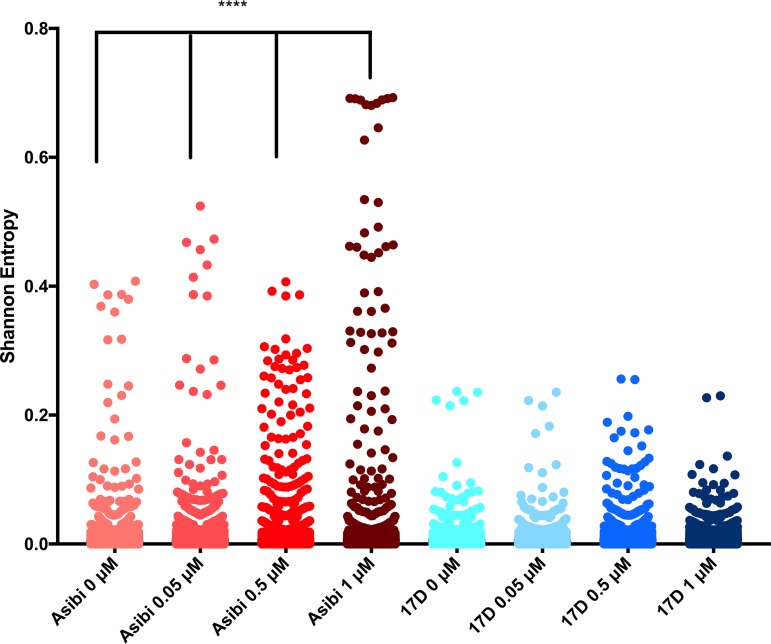
Entropy values for ribavirin-treated 17D-204 and Asibi viruses. Viruses were treated with the indicated concentrations of ribavirin, and RNA from these samples was sequenced by Illumina methods. All nucleotide positions, UTRs included, are depicted. Significance was detected in the average entropy of Asibi ribavirin-treated samples and the untreated sample (****, *P < *0.0001). No significant differences were detected between the average entropy of 17D-204 ribavirin-treated samples and the untreated sample (*P* > 0.999).

**FIG 4:**
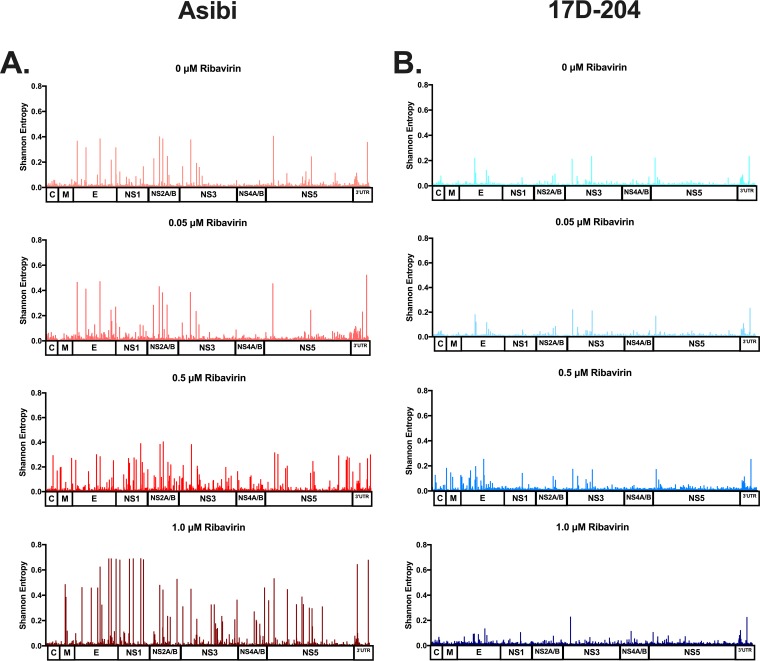
Entropy across entire genome mapped against variants detected. Viruses were treated with the indicated concentrations of ribavirin and RNA from these samples was sequenced by Illumina methods. (A and B) Entropy was calculated for each nucleotide across the genome for both Asibi (A) and 17D-204 (B).

**(i) WT Asibi versus 17D vaccine virus.** Four ribavirin treatment concentrations (0 μM, 0.05 μM, 0.5 μM, and 1 μM) were incubated with Asibi and 17D viruses, sampled at 48 h postinfection, and sequenced by massively parallel sequencing. Shannon’s entropy (34) was used to quantify the genetic diversity at each position in the YFV genome following treatment with ribavirin (Fig. 3). Shannon’s entropy measures the nucleotide complexity at each position in the genome and has been used most notably in studies of hepatitis virus diversity upon antiviral treatment (34, 35). The genome structure of the Asibi virus became more disordered with ribavirin as the average entropy of each Asibi sample differed significantly (Fig. 4A). Treatment with ≥0.5 μM ribavirin caused increases in entropy across the entire genome (Fig. 3 and 4A). Conversely, treatment of 17D virus with ribavirin had little effect on population structure, with the increase in average entropy over the entire genome being minimal and nonsignificant (Fig. 4B). Peaks of diversity were consistent between treated and untreated 17D virus samples.

**(ii) Effect of ribavirin on single nucleotide variant populations.** Genetic diversity was also evaluated in terms of single nucleotide variants (SNVs), defined as a nucleotide that differs from the consensus sequence by at least 1% of the population at that position.

**FIG 5:**
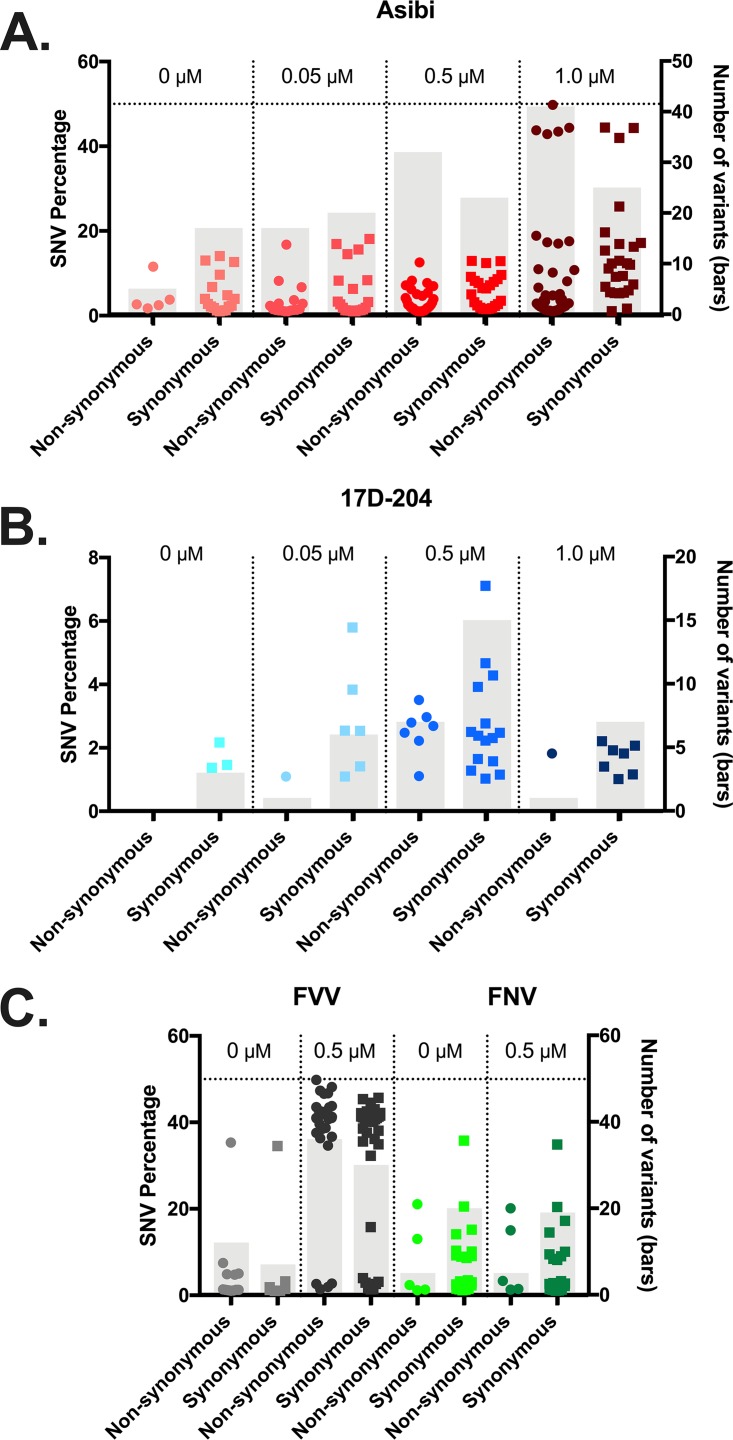
Variants detected in ribavirin-treated and untreated YFV samples. RNA sequenced by Illumina methods from four treatment concentrations. (A and C) V-Phaser2 v2.0 was used to detect synonymous (squares) and nonsynonymous (circles) variants in treated Asibi (A), 17D (B), and FVV/FNV (C). The total numbers of variants in both categories are represented by gray bars. Only variants that passed the strand bias test (*P < *0.05) and were detected above 1% are reported.

Treatment of Asibi virus with ribavirin resulted in a dose-dependent increase in both the number of SNVs and the number of nonsynonymous variants compared to synonymous variants. The 0.5 μM concentration caused the largest increase in genome variation (Fig. 5A). Twenty out of 26 SNVs detected in untreated samples could also be detected in treated samples, with the six remaining mutations being synonymous. In treated samples, the majority of new SNVs detected were nonsynonymous, indicating a response to the selection pressure posed by ribavirin. The diversity of Asibi virus NS genes showed larger increases in SNVs than with structural genes. In particular, the NS1, NS2A, and NS5 genes showed the greatest effect (see Table S1 in the supplemental material). With treatment, the frequency at which variants were detected increased significantly compared to that in untreated samples (*P* = 0.033) (Fig. 5A). With 1.0 μM treatment of Asibi virus, nine variants were detected near 50% frequency, of which five were located in the NS1 gene, three were located in the E gene, and one was found in the 3′-untranslated region (3′-UTR) (Table S1). Interestingly, four coding variants that differentiate 17D and Asibi viruses (E-380, E-407, NS1-307, and NS2A-118) were detected in ribavirin-treated Asibi samples (Table S1). The E-407 variant was detected in all treated Asibi virus samples sequenced, with a consensus change occurring in the 1.0 μM treatment.

10.1128/mBio.02294-19.1 TABLE S1 17D-204 treated with ribavirin generates less SNV than does ribavirin-treated Asibi. Variants were detected using V-Phaser2, and variants below 1% frequency and those that did not pass the strand bias test were discarded. Variants highlighted in green are common throughout the samples. Variants in red text are residues which distinguish 17D-204 and Asibi. Download Table S1, DOCX file, 0.1 MB. Copyright © 2019 Davis et al. 2019 Davis et al. This content is distributed under the terms of the Creative Commons Attribution 4.0 International license.

Although the number of SNVs in the 17D virus samples increased with increasing concentration of ribavirin, the frequency of variants detected remained low. The number of nonsynonymous SNVs was consistently lower than synonymous SNVs, suggesting that the virus was not responding to the selection pressure posed by ribavirin. In total, only seven nonsynonymous SNVs were detected across all ribavirin treatments sequenced, with each SNV being present in less than 5% of the population (Fig. 5B). Structural genes were most affected with treatment, with the majority of SNVs being detected in the E protein (Table S1). The increase in percentage of SNVs detected between treated 17D virus samples was not statistically significant (*P > *0.999).

**FIG 6:**
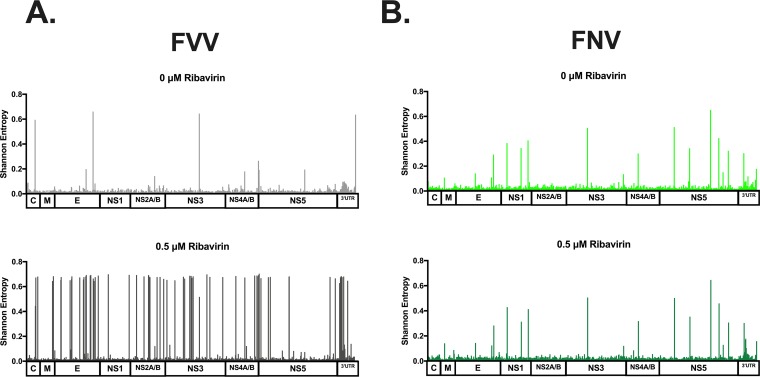
Entropy across entire genome of ribavirin-treated FNV virus and FVV. (A and B) RNA was isolated from treated and untreated FVV (A) and FNV virus (B) samples were sequenced by Illumina methods. Entropy was calculated for each nucleotide across the genome for both FVV (A) and FNV virus (B).

**(iii) NGS analysis of WT FVV versus FNV virus.** In order to confirm the resistance of vaccine strains to ribavirin treatment, untreated and 0.5 μM ribavirin FVV- and FNV virus-treated samples were sequenced by next-generation methods. As with Asibi virus, WT FVV experienced large increases in viral diversity by entropy (Fig. 6A), whereas the diversity of FNV virus was not affected by treatment with ribavirin (Fig. 6B). FVV displayed an increase in the number of SNVs detected in the ribavirin-treated sample (nonsynonymous SNVs, 30; synonymous SNVs, 36) compared to the untreated sample (nonsynonymous SNVs, 12; synonymous SNVs, 7) (Fig. 5C). SNVs detected in the ribavirin-treated FVV sample were detected at a significantly higher percentage than those detected in the untreated FVV sample (*P < *0.0001). Consistent with the 17D findings, the number of SNVs detected in ribavirin-treated (nonsynonymous SNVs, 5; synonymous SNVs, 20) and untreated (nonsynonymous SNVs, 5; synonymous SNVs, 19) FNV virus samples were consistent in both number and identity (Table S2).

10.1128/mBio.02294-19.2 TABLE S2 FNV virus treated with ribavirin generates less SNV than does ribavirin-treated FVV. Variants were detected using V-Phaser2, and variants below 1% frequency and those that did not pass the strand bias test were discarded. Variants highlighted in green are common throughout the samples. Download Table S2, DOCX file, 0.1 MB. Copyright © 2019 Davis et al. 2019 Davis et al. This content is distributed under the terms of the Creative Commons Attribution 4.0 International license.

**FIG 7:**
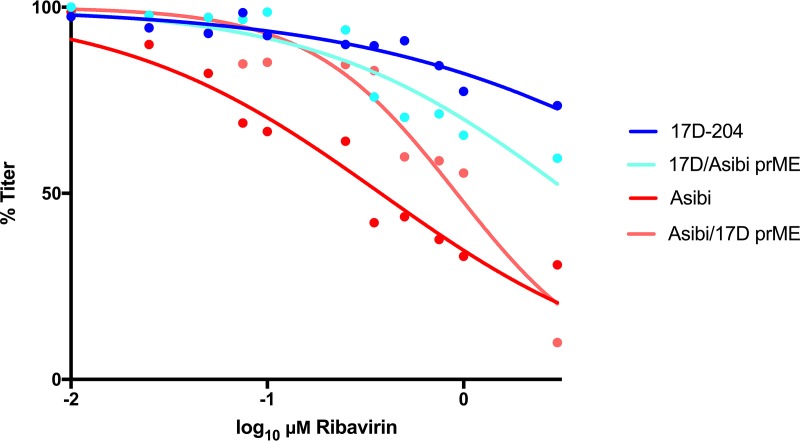
Dose response of 17D-204 and Asibi structural chimeras to *in vitro* ribavirin treatment. Infectious clone chimeras were constructed and incubated with the GTP nucleoside analog ribavirin in Vero cells. After 48 h, the supernatant was collected and titrated for viral load (FFU). The titers at each concentration were normalized to untreated, infected cells and fit using a dose-response nonlinear regression.

**(iv) Sensitivity to ribavirin is controlled by YFV nonstructural proteins.** To exclude virus passage history in the antiviral activity of ribavirin and determine if the YFV replication complex was involved in the resistance of 17D to ribavirin, infectious clone-derived viruses and structural chimeras (i.e., 17D infectious clone with Asibi virus prME genes or Asibi infectious clone with 17D virus prME genes; note that the capsid genes of Asibi and 17D viruses are identical in sequence) were generated using an infectious clone system (36, 37). The susceptibilities of 17D and Asibi infectious clone-derived viruses to ribavirin were compared to those of the parental noninfectious clone-derived viruses and were found to be not significantly different from the parental viruses used in the studies (*P > *0.999). Thus, the results of infectious-clone-derived viruses show that the effects of ribavirin were not due to the passage history of the viruses. The IC_50_ of the ribavirin-treated Asibi/17D prME virus (0.87 μM, *R*^2^ = 0.90) was similar to that of the Asibi virus infectious clone (0.37 μM, *R*^2^ = 0.94) (Fig. 7). The 17D/Asibi prME virus (19.43 μM, *R*^2^ = 0.68) was resistant to ribavirin, similar to the 17D virus (21.17 μM, *R*^2^ = 0.88) (Fig. 7), indicating that the structural proteins did not contribute to ribavirin resistance.

## DISCUSSION

Live-attenuated virus vaccines have been successfully used to combat infectious disease for decades, but due to their empirical derivation, little is known about their mechanisms of attenuation. This lack of understanding makes the development of next-generation live-attenuated vaccines difficult. The live-attenuated YF 17D vaccine virus has been used for 80 years to successfully reduce YF disease, and over 850 million doses of the vaccine have been distributed with exceptionally low levels of vaccine-associated adverse events (3). The success of the vaccine and availability of the parent virus, Asibi, make it an excellent model to understand the molecular basis of attenuation of a live-attenuated vaccine and the effects of viral diversity on vaccine function.

We have previously shown that the genetic diversity of 17D vaccine virus is low, whereas WT Asibi virus displays high levels of genetic diversity across the entire genome that is typical of an RNA virus. It has been proposed that this difference is due to an alteration of replication complex fidelity (20, 38). Previously, resistance to ribavirin has been shown to be a property of high-fidelity replication complexes in other RNA viruses, such as foot-and-mouth disease virus and poliovirus (8, 39). The dual mechanism of action for ribavirin contributes to lethal mutagenesis of RNA viruses, as (i) low-fidelity viral RdRps incorporate ribavirin into nascent genomes and (ii) available GTP declines, resulting in increased frequency of nucleotide mismatch or the halting of replication all together. The susceptibility of WT YFV strains to ribavirin (IC_50_ values, 0.37 μM and 0.51 μM for Asibi and FVV, respectively) is consistent with findings from studies of other RNA viruses encoding a low-fidelity RdRp, such as Lassa and Crimean-Congo hemorrhagic fever viruses (0.3 μM and 0.5 μM *in vitro*, respectively) (28, 40, 41). The IC_50_s of ribavirin-treated 17D virus and FNV virus are far above concentrations those considered effective based on toxicity *in vitro*, suggesting that the drug is ineffective against these strains (42). It has been suggested that a speed/fidelity trade-off is responsible for low fecundity of RNA virus RdRps, as faster replicators can better avoid the immune response and spread through the host (43). In order to test the contribution of the anti-IMPDH activity of ribavirin on the YFV, the antiviral drug MPA was utilized. MPA is an antiviral whose mechanism exclusively inhibits IMPDH. This drug has been shown to be highly effective against 17D but had never been tested against any WT YFV strain (31, 32). The current study confirmed that WT Asibi virus and FVV were susceptible to MPA at a similar IC_50_ to 17D and FNV (Fig. 2). Together, this suggests that it is the NTP activity of ribavirin that influences the difference in susceptibility between WT and vaccine YFV, rather than the GTP depletion (Fig. 2).

Ribavirin has been shown to target NS3 (serine protease, helicase, and nucleotide triphosphatase) of hepatitis C virus, dengue virus NS5 methyltransferase, poliovirus RdRp, and chikungunya virus nsP4, which contains the RdRp (8, 19, 44, 45). Consistent with these findings, experiments utilizing chimeric viruses suggest that the Asibi virus replication complex (NS1 to NS5), or a component of the complex, confers sensitivity to the NTP activity of the drug (Fig. 7). Both structural and nonstructural genes increased in diversity after the treatment of Asibi virus, though 81% of SNVs were detected within the replication complex genes, displaying the importance of the NS proteins in ribavirin activity. In particular, the Asibi virus NS5 gene was highly affected by treatment with ribavirin by both diversity and SNV indices. The majority (56%) of SNVs detected in this gene after treatment were nonsynonymous and within the palm and thumb domains of the viral RdRp. These domains of flavivirus RdRps control priming and polymerase activities (46, 47). In general, the number of nonsynonymous mutations in ribavirin-treated samples increased with increasing ribavirin concentration, with the number of SNVs resulting in a coding change outnumbering silent SNVs from 0.5 μM ribavirin onward. The increase in nonsynonymous mutations is suggestive of a response to a selection pressure, and the emergence of localized NS mutants likely demonstrates the interaction of NS proteins with the compound. This coupled with a dramatic decrease in infectivity titer suggests that the NTP activity of ribavirin was causing the virus to enter error catastrophe. The strain of Asibi virus used in this study had been passaged previously in C6/36 cells and rhesus macaques. These serial passages could have conceivably impacted the genome structure of the virus and consequent sensitivity to ribavirin. We do not believe this is the case, as the full-length Asibi infectious clone virus had a response statistically indistinguishable from that of the drug compared to the biological virus. The susceptibility and genetic degradation of WT YFV after ribavirin treatment were confirmed with the WT strain FVV. FVV had am IC_50_ similar to that of Asibi virus and had increases in diversity and SNVs, of which 66% were detected in the replication complex. Our studies indicate that ribavirin is being incorporated into the genome of WT YFV and should be tested as a treatment for YFV infection. This warrants further investigation, as most studies to date have utilized 17D vaccine virus as a model system, which is not representative of WT YFV strains (48, 49).

Little variation was detected in the genotype of ribavirin-treated YFV vaccine strains, and only a negligible decrease in infectivity titer was observed, suggesting a resistance to the lethal mutagenesis of ribavirin by YFV vaccine strains. This confirms previous reports examining the activity of ribavirin against 17D virus (31). In comparison to ribavirin-treated Asibi virus samples, the NS genes of 17D virus maintained low levels of diversity across all ribavirin concentrations tested. SNVs detected in NS genes were synonymous and reported at <5% frequency, with one exception of a nonsynonymous mutation in the 0.05 μM treatment of 17D (NS3-V195I; 1.1%). NS5 remained low in diversity, and only one synonymous SNV (NS5-314; 1.4%) was detected in the NS5 gene of ribavirin-treated 17D samples. The genetic stability of 17D genes encoding enzymatic activities and the insignificant change in infectivity titer following treatment suggest that the replication complex of the vaccine viral genome as a whole is stable and resistant to the genetic pressure exerted by ribavirin. Using FNV virus, which was derived from WT FVV, this resistance and genomic stability were confirmed in another vaccine strain of YFV. As 17D and FNV virus vaccines are both resistant to ribavirin, it can be concluded that the empirical derivation of both vaccines developed a replication complex that excludes the NTP.

It is possible that the extensive serial passaging of WT Asibi virus and FVV (176 and 128 passages, respectively) caused the WT virus to adapt to an unchanging environment where an unwavering consensus sequence was the most fit. This adaptation caused the virus to be less fit in a biological environment where the vaccines could no longer respond to selection pressure (i.e., multiplication in the liver and mosquitoes) (50). Resistance to the genetic pressure of an NTP antiviral is an attractive quality for a live-attenuated vaccine. This resistance could be, in part, a mechanism of attenuation and stability that confers the impressive safety record of the 17D vaccine. Indeed, we have previously shown that 17D vaccine strain accumulates very few mutations once administered (51). Although FNV virus is resistant to ribavirin and less diverse than FVV, it is both (i) more diverse and (ii) more sensitive to ribavirin treatment than is the 17D virus. This has been shown previously (7, 52) and may in part explain the increase in adverse events associated with FNV virus when used in children, as FNV virus is less able to maintain genetic integrity, resulting in more frequent reversions to WT.

In this study, the effects of ribavirin on two WT YFV strains and the two LAV strains derived from them were compared under a hypothesis that an acquired fidelity mechanism contributes to the attenuation of an empirical flavivirus vaccine. It had been previously shown that 17D was resistant to the drug; however, the WT Asibi strain was not tested in a similar context, which was performed herein (31). The WT viruses, Asibi and FVV, were found to be highly sensitive to ribavirin, whereas the 17D and FNV virus vaccine strains were not. The findings support the hypothesis that the molecular basis of attenuation of YFV vaccine is due, at least in part, to the inability of the vaccine viruses to revert to virulence; the principal evidence for this was by the lack of diversity following the genetic pressure exerted by ribavirin treatment. Whether or not the same mechanism is utilized by other LAVs remains to be determined. However, studies on molecular basis of attenuation of live-attenuated poliovirus vaccines have shown that the 5′UTR and capsid confer the attenuated phenotype (8, 53). Thus, the mechanism of fidelity attenuation of the YFV 17D vaccine differs from that of other vaccine strains in a manner exclusive to flavivirus replication. The findings are useful in the development of other currently utilized live-attenuated viral vaccines and contribute to the use of a quasispecies framework in vaccine characterization. Overall, the findings indicate that the attenuation of YFV live vaccines is due, in part, to the genetic homogeneity of the attenuated strains.

## MATERIALS AND METHODS

### Antivirals and viral stocks.

Ribavirin (lot no. M611989; Cadila Healthcare Pharmaceuticals) was reconstituted in high-performance liquid chromatography (HPLC)-grade water and sterilized using a 0.2-μm filter. A 10 μM working solution was then obtained by diluting the stock in minimal essential medium (MEM) supplemented with 2% fetal bovine serum (FBS). This working stock was used to make ribavirin concentrations of 6 μM, 2 μM, 1 μM, 0.7 μM, 0.5 μM, 0.2 μM, 0.15 μM, 0.1 μM, 0.05 μM, and 0.02 μM for the antiviral assays.

MPA (brand CellCept, lot no. M3916; Roche) was reconstituted at 25 mM in HPLC-grade water. A 1 mM working solution was obtained by diluting the stock in MEM supplemented with 2% FBS. This working stock was used to make a dose-response curve of 2-fold dilutions from MPA concentrations of 1 mM to 0.24 μM.

The 17D-204 virus used was reconstituted directly from a dose of commercial YF-Vax vaccine (lot no. UF795AA; Sanofi-Pasteur). The Asibi virus used was obtained from the World Reference Center for Emerging Viruses and Arboviruses (WRCEVA, Galveston, TX; GenBank accession no. KF769016) and is the lowest passage number available, namely, six passages in rhesus macaques and 3 passages in C6/36 cells. FVV (GenBank accession no. U21056) and FNV-Yale (GenBank accession no. U21055) viruses used were received from the WRCEVA and passaged once in Vero cells (54).

Chimeric 17D-204 and Asibi viruses with prM and E (prME) genes swapped were constructed using infectious clone systems (36, 55). Briefly, prME genes from 17D-204 and Asibi viruses were exchanged (QuikChange XL; Agilent Technologies). RNA was transcribed *in vitro* in the presence of 5′-cap analogue and electroporated into Vero cells (AmpliCap SP6 message maker; CellScript). Virus was harvested at 80% cytopathic effect (CPE) and sequenced by Illumina methods to confirm sequence identity (University of Texas Medical Branch [UTMB] Sequencing Core).

All viral infectivity assays were undertaken in biosafety level 3 (BSL3) facilities at the University of Texas Medical Branch.

### Viral infectivity reduction assay.

As described by Leyssen et al. (31), viral infectivity reduction assays were performed. Briefly, Vero monkey kidney cells were seeded at 80% confluence in 12-well plates and were infected at a multiplicity of infection (MOI) of 0.05 at room temperature for 30 min. Ribavirin or MPA was added at the concentrations stated above in a volume of 750 μl. Following incubation at 37°C for 1 h, another 750 μl MEM supplemented with 2% FBS was added to each well to reach the desired concentration. Virus-containing culture medium was harvested at 48 h postinfection. Viral titers were determined by a focus-forming assay and immunostained using anti-Asibi mouse immune ascites fluid (WRCEVA), followed by biotinylated goat anti-mouse IgG and NeutrAvidin conjugated with horseradish peroxidase (HRP; Thermo Fisher). Each assay was undertaken in triplicate.

### Next-generation sequencing.

Viral RNA was extracted using a QIAamp viral RNA isolation kit and sequenced by the UTMB Sequencing Core. cDNA libraries were prepared using random hexamer priming (TruSeq RNA library prep kit) and sequenced on an Illumina HiSeq 1500 platform. Reads were trimmed of adapter sequences and quality controlled for quality scores greater than 30 (Trimmomatic). The reads were aligned to a reference genome (GenBank accession no. AY640589) using Bowtie2 with paired-end, very sensitive local parameters. PCR duplicates were removed with MarkDuplicates (Picard Tools; Broad Institute, MIT). All samples were downsampled to the lowest mean coverage of the data set, 9,984 reads per base. Shannon’s entropy was calculated using custom R scripts over possible nucleotide variables (A, U, C, G, −) using the method described by Nishijima et al (34). Variants were called using default settings of the software V-Phaser2 (v2.0) (56). Any variants that did not pass the strand bias test were removed from further analysis.

### Statistical analyses.

All statistical analyses were completed in the GraphPad Prism (v7.2.1). A one-way analysis of variance (ANOVA) was performed with Friedman’s *post hoc* multiple-comparison test (*P < *0.05) to determine antiviral 50% inhibitory concentrations (IC_50_) values and significant differences between four-parameter nonlinear fits of sensitivity curves. A one-way ANOVA was performed with Friedman’s *post hoc* multiple-comparison test (*P < *0.05) to determine significant differences in average entropy across viruses and treatments. A one-way ANOVA was performed with Kruskal-Wallis *post hoc* multiple-comparison test (*P < *0.05) to determine significant differences in the frequencies of variants.
