# Supplementary material for: Attenuation of Live-Attenuated Yellow Fever 17D Vaccine Virus Is Localized to a High-Fidelity Replication Complex
Source: mBio. 2019 Oct 22;10(5):e02294-19. doi: 10.1128/mBio.02294-19 (PMC6805994; doi:10.1128/mBio.02294-19)
Supplement: TABLE S1 [file mBio.02294-19-st001.docx]

**Supplementary table 1: 17D-204 treated with ribavirin generates less SNV than ribavirin Asibi.** Variants were detected using VPhaserII with variants below 1% frequency and those that did not pass the strand bias test were discarded. Variants highlighted in green are common throughout samples. Variants in red text are residues which distinguish 17D-204 and Asibi.

| 17D 0 uM |  |  |  |  |  |  |  |  |  |  |  |
| --- | --- | --- | --- | --- | --- | --- | --- | --- | --- | --- | --- |
| CDS Position | Consensus | Variant | Protein Position | Consensus | Variant | Gene | Codon within Gene | Nucleotide | Amino Acid | Shannon Entropy | SNV Percentage |
| 1345 | C | T | 449 | L | * | E | 286 | C1345T | L286* | 0.10 | 2.17 |
| 3936 | T | C | 1312 | N | * | NS2A | 182 | T3936C | N182* | 0.08 | 1.46 |
| 7173 | A | G | 2391 | A | C | NS4B | 135 | A7173G | A135C | 0.07 | 1.37 |
|  |  |  |  |  |  |  |  |  |  |  |  |
| 17D 0.05 uM |  |  |  |  |  |  |  |  |  |  |  |
| CDS Position | Consensus | Variant | Protein Position | Consensus | Variant | Gene | Codon within Gene | Nucleotide | Amino Acid | Shannon Entopy | SNV Percentage |
| 1345 | C | T | 449 | L | * | E | 286 | C1345T | L286* | 0.12 | 2.53 |
| 1701 | T | C | 567 | S | * | E | 404 | T1701C | S404* | 0.12 | 2.54 |
| 3936 | T | C | 1312 | N | * | NS2A | 182 | T3936C | N182* | 0.07 | 1.42 |
| 4590 | G | A | 1530 | G | * | NS3 | 46 | G4590A | G46* | 0.22 | 5.79 |
| 5035 | G | A | 1679 | V | I | NS3 | 195 | G5035A | V195I | 0.05 | 1.10 |
| 7378 | C | T | 2460 | L | * | NS4B | 204 | C7378T | L204* | 0.17 | 3.84 |
|  |  |  |  |  |  |  |  |  |  |  |  |
| 17D 0.5 uM |  |  |  |  |  |  |  |  |  |  |  |
| CDS Position | Consensus | Variant | Protein Position | Consensus | Variant | Gene | Codon within Gene | Nucleotide | Amino Acid | Shannon Entropy | SNV Percentage |
| -6 | C | T | - | - | - | 5'UTR | - | - | - | 0.13 | 2.97 |
| 27 | A | G | 9 | K | * | C | 9 | A27G | K9* | 0.07 | 1.28 |
| 370 | T | C | 124 | L | * | M | 3 | T370C | L3* | 0.19 | 4.67 |
| 511 | C | T | 171 | P | S | M | 50 | C511T | P50S | 0.15 | 3.51 |
| 576 | T | C | 192 | V | * | M | 71 | T576C | V71* | 0.12 | 2.50 |
| 848 | C | T | 283 | A | V | M | 162 | C848T | A162V | 0.12 | 2.69 |
| 852 | C | T | 284 | Y | * | M | 163 | C852T | Y163* | 0.13 | 2.77 |
| 861 | C | T | 287 | H | * | E | 2 | C861T | H2* | 0.12 | 2.48 |
| 885 | T | C | 295 | D | * | E | 10 | T885C | D10* | 0.11 | 2.38 |
| 938 | A | G | 313 | D | G | E | 28 | A938G | D28G | 0.06 | 1.11 |
| 1127 | C | T | 376 | A | V | E | 91 | C1127T | A91V | 0.11 | 2.22 |
| 1161 | C | T | 387 | G | * | E | 102 | C1161T | G102* | 0.10 | 2.23 |
| 1173 | C | T | 391 | G | * | E | 106 | C1173T | G106* | 0.16 | 3.92 |
| 1388 | C | T | 463 | A | V | E | 178 | C1388T | A178V | 0.12 | 2.48 |
| 1521 | C | T | 507 | G | * | E | 222 | C1521T | G222* | 0.08 | 1.65 |
| 1614 | C | T | 538 | S | * | E | 253 | C1614T | S253* | 0.26 | 7.11 |
| 1628 | T | C | 543 | L | P | E | 258 | T1628C | L258P | 0.13 | 2.80 |
| 1905 | T | C | 635 | V | * | E | 350 | T1905C | V350* | 0.06 | 1.16 |
| 2904 | A | G | 968 | G | * | NS1 | 190 | A2904G | G190* | 0.14 | 1.58 |
| 3936 | T | C | 1312 | N | * | NS2A | 182 | T3936C | N182* | 0.12 | 2.31 |
| 4590 | G | A | 1530 | G | * | NS3 | 46 | G4590A | G46* | 0.18 | 4.28 |
| 5079 | C | A | 1693 | L | * | NS3 | 209 | C5079A | L209* | 0.06 | 1.03 |
|  |  |  |  |  |  |  |  |  |  |  |  |
| 17D 1.0 uM |  |  |  |  |  |  |  |  |  |  |  |
| CDS Position | Consensus | Variant | Protein Position | Consensus | Variant | Gene | Codon within Gene | Nucleotide | Amino Acid | Shannon Entropy | SNV Percentage |
| 1345 | C | T | 449 | L | * | E | 164 | C1345T | L164* | 0.10 | 1.92 |
| 1484 | C | T | 495 | A | V | E | 210 | C1484T | A210V | 0.09 | 1.83 |
| 5904 | A | G | 1968 | E | * | NS3 | 484 | A5904G | E484* | 0.06 | 1.16 |
| 6636 | C | A | 2212 | S | * | NS4A | 105 | C6636A | S105* | 0.12 | 2.21 |
| 6748 | C | T | 2250 | L | * | NS4A | 143 | C6748T | L143* | 0.06 | 1.02 |
| 7378 | C | T | 2460 | L | * | NS4B | 204 | C7378T | L204* | 0.11 | 2.07 |
| 8460 | C | T | 2820 | T | * | NS5 | 314 | C8460T | T314* | 0.07 | 1.41 |
|  |  |  |  |  |  |  |  |  |  |  |  |
| Asibi 0 uM |  |  |  |  |  |  |  |  |  |  |  |
| CDS Position | Consensus | Variant | Protein Position | Consensus | Variant | Gene | Codon within Gene | Nucleotide | Amino Acid | Shannon Entropy | SNV Percentage |
| 936 | A | C | 312 | Q | H | E | 27 | A936C | Q27H | 0.37 | 11.58 |
| 1227 | C | T | 409 | S | * | E | 124 | C1227T | S124* | 0.32 | 9.68 |
| 1611 | C | T | 537 | G | * | E | 252 | C1611T | G252* | 0.10 | 2.12 |
| 1701 | C | T | 567 | S | * | E | 282 | C1701T | S282* | 0.39 | 13.04 |
| 1875 | T | C | 625 | L | * | E | 340 | T1875C | L340* | 0.07 | 1.23 |
| 2238 | C | T | 746 | L | * | E | 461 | C2238T | L461* | 0.10 | 2.17 |
| 2363 | A | G | 788 | K | R | NS1 | 10 | A2363G | K10R | 0.13 | 2.70 |
| 2673 | T | C | 891 | Y | * | NS1 | 113 | T2673C | Y113* | 0.07 | 1.28 |
| 3053 | A | C | 1018 | E | A | NS1 | 240 | A3053C | E240A | 0.07 | 1.18 |
| 3156 | A | G | 1052 | E | * | NS1 | 274 | A3156G | E274* | 0.17 | 3.97 |
| 3963 | T | C | 1231 | T | * | NS2A | 101 | T3963C | T101* | 0.25 | 6.78 |
| 4473 | T | C | 1491 | D | * | NS3 | 7 | T4473C | D7* | 0.17 | 3.99 |
| 4746 | A | G | 1582 | Q | * | NS3 | 98 | A4746G | Q98* | 0.38 | 12.66 |
| 4941 | C | T | 1647 | S | * | NS3 | 163 | C4941T | S163* | 0.19 | 4.83 |
| 5035 | A | G | 1679 | I | V | NS3 | 195 | A5035G | I195V | 0.16 | 3.76 |
| 5130 | G | A | 1710 | L | * | NS3 | 226 | G5130A | L226* | 0.09 | 1.83 |
| 7057 | C | T | 2353 | L | * | NS4B | 97 | C7057T | L97* | 0.06 | 1.03 |
| 7524 | T | C | 2508 | S | * | NS5 | 2 | T7524C | S2* | 0.41 | 14.06 |
| 7974 | G | A | 2658 | S | * | NS5 | 152 | G7974A | S152* | 0.11 | 2.37 |
| 8500 | A | G | 2834 | I | V | NS5 | 328 | A8500G | I328V | 0.09 | 1.72 |
| 8616 | T | C | 2872 | D | * | NS5 | 366 | T8616C | D366* | 0.13 | 2.78 |
| 8724 | A | G | 2908 | A | * | NS5 | 402 | A8724G | A402* | 0.00 | 1.18 |
| 9598 | G | A | 3200 | D | N | NS5 | 694 | G9598A | D694N | 0.12 | 2.46 |
| 10314 | T | A | - | - | - | 3'UTR | - | T10314A | - | 0.12 | 1.84 |
| 10319 | C | T | - | - | - | 3'UTR | - | C10319T | - | 0.06 | 1.04 |
| 10682 | G | A | - | - | - | 3'UTR | - | G10682A | - | 0.36 | 11.64 |
|  |  |  |  |  |  |  |  |  |  |  |  |
| Asibi 0.05 uM |  |  |  |  |  |  |  |  |  |  |  |
| CDS Position | Consensus | Variant | Protein Position | Consensus | Variant | Gene | Codon within Gene | Nucleotide | Amino Acid | Mutational Freq | SNV Percentage |
| -36 | T | C | - | - | - | 5'UTR | - | - | - | 0.07 | 1.21 |
| 62 | G | A | 21 | R | H | C | 21 | G62A | R21H | 0.07 | 1.30 |
| 936 | A | C | 312 | Q | H | E | 27 | A936C | Q27H | 0.47 | 16.77 |
| 994 | A | G | 332 | T | A | E | 47 | A994G | T47A | 0.07 | 1.25 |
| 1227 | C | T | 409 | S | * | E | 124 | C1227T | S124* | 0.00 | 14.50 |
| 1237 | T | C | 413 | F | L | E | 128 | T1237C | F128L | 0.06 | 1.01 |
| 1320 | C | T | 440 | D | * | E | 155 | C1320T | D155* | 0.09 | 1.86 |
| 1431 | C | T | 477 | N | * | E | 192 | C1431T | N192* | 0.06 | 1.18 |
| 1701 | C | T | 567 | S | * | E | 282 | C1701T | S282* | 0.47 | 18.12 |
| 1994 | C | G | 665 | T | R | E | 380 | C1994G | T380R | 0.08 | 1.60 |
| 2075 | C | T | 692 | A | V | E | 407 | C2075T | A407V | 0.25 | 6.71 |
| 2101 | G | A | 701 | A | T | E | 416 | G2101A | A416T | 0.16 | 3.67 |
| 2231 | C | T | 744 | A | V | E | 459 | C2231T | A459V | 0.27 | 8.24 |
| 2238 | C | T | 746 | L | * | E | 461 | C2238T | L461* | 0.14 | 3.22 |
| 2363 | A | G | 788 | K | R | NS1 | 10 | A2363G | K10R | 0.11 | 2.31 |
| 2569 | C | T | 857 | L | F | NS1 | 79 | C2569T | L79F | 0.07 | 1.21 |
| 2586 | G | A | 862 | V | * | NS1 | 84 | G2586A | V84* | 0.06 | 1.04 |
| 2673 | T | C | 891 | Y | * | NS1 | 113 | T2673C | Y113* | 0.06 | 1.04 |
| 3053 | A | C | 1018 | E | A | NS1 | 240 | A3053C | E240A | 0.13 | 2.82 |
| 3156 | A | G | 1052 | E | * | NS1 | 274 | A3156G | E274* | 0.12 | 2.69 |
| 3253 | A | G | 1085 | I | V | NS1 | 307 | A3253G | I307V | 0.08 | 1.51 |
| 3504 | G | A | 1168 | L | * | NS2A | 38 | G3504A | L38* | 0.29 | 8.28 |
| 3699 | A | G | 1233 | V | * | NS2A | 103 | A3699G | V103* | 0.00 | 15.60 |
| 3867 | T | C | 1289 | T | * | NS2A | 159 | T3867C | T159* | 0.09 | 1.87 |
| 3963 | T | C | 1321 | T | * | NS2A | 191 | T3963C | T191* | 0.29 | 8.38 |
| 4473 | T | C | 1491 | D | * | NS3 | 7 | T4473C | D7* | 0.15 | 3.32 |
| 4941 | C | T | 1647 | S | * | NS3 | 163 | C4941T | S163* | 0.24 | 6.34 |
| 5035 | A | G | 1679 | I | V | NS3 | 195 | A5035G | I195V | 0.13 | 2.89 |
| 5391 | A | G | 1797 | E | * | NS3 | 313 | A5391G | E313* | 0.07 | 1.27 |
| 7287 | A | G | 2429 | L | * | NS4B | 173 | A7287G | L173* | 0.07 | 1.27 |
| 7524 | T | C | 2508 | S | * | NS5 | 2 | T7524C | S2* | 0.46 | 16.93 |
| 8616 | T | C | 2872 | D | * | NS5 | 366 | T8616C | D366* | 0.07 | 1.22 |
| 9053 | G | A | 3018 | G | D | NS5 | 512 | G9053A | G512D | 0.06 | 1.03 |
| 9534 | G | A | 3178 | R | * | NS5 | 672 | G9534A | R672* | 0.08 | 1.50 |
| 9598 | G | A | 3200 | D | N | NS5 | 694 | G9598A | D694N | 0.07 | 1.20 |
| 9704 | T | C | 3235 | V | A | NS5 | 729 | T9704C | V729A | 0.07 | 1.36 |
| 10127 | T | C | 3376 | V | A | NS5 | 870 | T10127C | V870A | 0.07 | 1.34 |
| 10167 | T | C | 3389 | Y | * | NS5 | 883 | T10167C | Y883* | 0.07 | 1.21 |
| 10239 | C | T | - | - | - | 3'UTR | - | C10239T | - | 0.06 | 1.03 |
| 10265 | A | G | - | - | - | 3'UTR | - | A10265G | - | 0.08 | 1.59 |
| 10472 | A | T | - | - | - | 3'UTR | - | A10472T | - | 0.10 | 1.82 |
|  |  |  |  |  |  |  |  |  |  |  |  |
| Asibi 0.5 uM |  |  |  |  |  |  |  |  |  |  |  |
| CDS Position | Consensus | Variant | Protein Position | Consensus | Variant | Gene | Codon within Gene | Nucleotide | Amino Acid | Mutational Freq | SNV Percentage |
| 266 | C | T | 89 | A | V | C | 89 | C266T | A89V | 0.17 | 4.16 |
| 368 | C | T | 123 | T | I | M | 2 | C368T | T2I | 0.20 | 5.10 |
| 554 | G | A | 185 | W | * | M | 64 | G554A | W64* | 0.08 | 1.63 |
| 781 | T | C | 261 | Y | H | M | 140 | T781C | Y140H | 0.07 | 1.21 |
| 888 | C | T | 296 | F | * | E | 11 | C888T | F11* | 0.26 | 7.18 |
| 1312 | A | G | 438 | N | D | E | 153 | A1312G | N153D | 0.17 | 3.94 |
| 1331 | T | C | 444 | L | P | E | 159 | T1331C | L159P | 0.06 | 1.16 |
| 1925 | C | T | 642 | S | L | E | 357 | C1925T | S357L | 0.08 | 1.48 |
| 2007 | T | C | 669 | R | * | E | 384 | T2007C | R384* | 0.10 | 1.88 |
| 2091 | C | T | 697 | I | * | E | 412 | C2091T | I412* | 0.12 | 2.45 |
| 2144 | T | C | 715 | V | A | E | 430 | T2144C | V430A | 0.25 | 6.99 |
| 2487 | A | G | 829 | E | * | NS1 | 51 | A2487G | E51* | 0.14 | 3.17 |
| 2629 | A | G | 877 | R | G | NS1 | 99 | A2629G | R99G | 0.06 | 1.05 |
| 2649 | C | T | 883 | S | * | NS1 | 105 | C2649T | S105* | 0.27 | 7.74 |
| 2679 | G | T | 893 | W | C | NS1 | 115 | G2679T | W115C | 0.23 | 6.24 |
| 2804 | A | G | 935 | E | G | NS1 | 157 | A2804G | E157G | 0.09 | 1.84 |
| 2832 | C | T | 944 | R | * | NS1 | 166 | C2832T | R166* | 0.28 | 7.96 |
| 3053 | A | C | 1018 | E | A | NS1 | 240 | A3053C | E240A | 0.39 | 12.58 |
| 3061 | A | G | 1021 | M | V | NS1 | 243 | A3061G | M243V | 0.06 | 1.03 |
| 3108 | T | C | 1036 | P | * | NS1 | 258 | T3108C | P258* | 0.24 | 6.50 |
| 3161 | A | G | 1054 | K | R | NS1 | 276 | A3161G | K276R | 0.15 | 3.58 |
| 3699 | A | G | 1233 | V | * | NS2A | 103 | A3699G | V103* | 0.39 | 12.89 |
| 3700 | C | T | 1234 | L | * | NS2A | 104 | C3700T | L104* | 0.28 | 8.22 |
| 3743 | T | C | 1248 | M | T | NS2A | 118 | T3743C | M118T | 0.12 | 2.57 |
| 3807 | A | T | 1269 | V | * | NS2A | 139 | A3807T | V139* | 0.41 | 12.43 |
| 3963 | T | C | 1321 | T | * | NS2A | 191 | T3963C | T191* | 0.24 | 6.45 |
| 4174 | C | T | 1392 | L | * | NS2B | 38 | C4174T | L38* | 0.11 | 2.26 |
| 4248 | A | G | 1416 | E | * | NS2B | 62 | A4248G | E62* | 0.08 | 1.61 |
| 4356 | G | A | 1452 | V | * | NS2B | 98 | G4356A | V98* | 0.13 | 2.74 |
| 4421 | G | A | 1474 | G | E | NS2B | 120 | G4421A | G120E | 0.00 | 2.23 |
| 4682 | A | G | 1561 | D | G | NS3 | 77 | A4682G | D77G | 0.06 | 1.00 |
| 4709 | A | G | 1570 | K | R | NS3 | 86 | A4709G | K86R | 0.06 | 1.00 |
| 4746 | A | G | 1582 | Q | * | NS3 | 98 | A4746G | Q98* | 0.38 | 12.91 |
| 4979 | A | G | 1660 | E | G | NS3 | 176 | A4979G | E176G | 0.06 | 1.02 |
| 5006 | C | T | 1669 | P | L | NS3 | 185 | C5006T | P185L | 0.14 | 3.12 |
| 5035 | A | G | 1679 | I | V | NS3 | 195 | A5035G | I195V | 0.10 | 1.96 |
| 5469 | T | C | 1823 | D | * | NS3 | 339 | T5469C | D339* | 0.09 | 1.79 |
| 5484 | A | G | 1828 | I | M | NS3 | 344 | A5484G | I344M | 0.09 | 1.83 |
| 5626 | A | G | 1876 | R | G | NS3 | 392 | A5626G | R392G | 0.07 | 1.36 |
| 5895 | T | C | 1965 | P | * | NS3 | 481 | T5895C | P481* | 0.20 | 5.05 |
| 5936 | C | T | 1979 | A | V | NS3 | 495 | C5936T | A495V | 0.09 | 1.72 |
| 5992 | G | A | 1998 | G | S | NS3 | 514 | G5992A | G514S | 0.11 | 2.36 |
| 6326 | C | T | 2109 | A | V | NS4A | 2 | C6326T | A2V | 0.09 | 1.94 |
| 6704 | T | C | 2235 | I | T | NS4A | 128 | T6704C | I128T | 0.12 | 2.55 |
| 7302 | T | C | 2434 | A | * | NS4B | 178 | T7302C | A178* | 0.08 | 1.54 |
| 7524 | T | C | 2508 | S | * | NS5 | 2 | T7524C | S2* | 0.00 | 9.63 |
| 7632 | T | C | 2544 | D | * | NS5 | 38 | T7632C | D38* | 0.31 | 9.17 |
| 7793 | C | T | 2598 | A | V | NS5 | 92 | C7793T | A92V | 0.07 | 1.22 |
| 7889 | T | C | 2630 | I | T | NS5 | 124 | T7889C | I124T | 0.19 | 4.66 |
| 7945 | C | T | 2649 | L | F | NS5 | 143 | C7945T | L143F | 0.21 | 5.42 |
| 8724 | A | G | 2908 | A | * | NS5 | 402 | A8724G | A402* | 0.15 | 3.53 |
| 9660 | C | T | 3220 | S | * | NS5 | 714 | C9660T | S714* | 0.29 | 8.60 |
| 9913 | T | C | 3305 | W | R | NS5 | 799 | T9913C | W799R | 0.26 | 7.16 |
| 9947 | C | A | 3316 | T | K | NS5 | 810 | C9947A | T810K | 0.29 | 8.28 |
| 10012 | A | G | 3338 | T | A | NS5 | 832 | A10012G | T832A | 0.28 | 7.73 |
| 10134 | T | C | - | - | - | 3'UTR | - | T10134C | - | 0.16 | 3.84 |
| 10607 | A | G | - | - | - | 3'UTR | - | A10607G | - | 0.10 | 2.05 |
| 10624 | T | C | - | - | - | 3'UTR | - | T10624C | - | 0.27 | 7.58 |
| 10719 | C | T | - | - | - | 3'UTR | - | C10719T | - | 0.30 | 8.72 |
|  |  |  |  |  |  |  |  |  |  |  |  |
| Asibi 1 uM |  |  |  |  |  |  |  |  |  |  |  |
| CDS Position | Consensus | Variant | Protein Position | Consensus | Variant | Gene | Codon within Gene | Nucleotide | Amino Acid | Mutational Freq | SNV Percentage |
| 534 | G | A | 178 | E | * | M | 57 | G534A | E57* | 0.49 | 16.92 |
| 558 | C | T | 186 | C | * | M | 65 | C558T | C65* | 0.39 | 12.36 |
| 601 | T | C | 201 | S | P | M | 80 | T601C | S80P | 0.12 | 3.36 |
| 884 | A | G | 295 | D | G | M | 174 | A884G | D174G | 0.10 | 2.78 |
| 1094 | C | T | 365 | A | V | E | 80 | C1094T | A80V | 0.46 | 18.87 |
| 1405 | G | A | 469 | V | M | E | 184 | G1405A | V184M | 0.01 | 17.33 |
| 1622 | C | T | 541 | T | I | E | 256 | C1622T | T256I | 0.46 | 17.55 |
| 1781 | A | G | 594 | N | S | E | 309 | A1781G | N309S | 0.06 | 1.33 |
| 1992 | G | A | 664 | G | * | E | 379 | G1992A | G379* | 0.69 | 44.32 |
| 2048 | A | G | 683 | K | R | E | 398 | A2048G | K398R | 0.08 | 1.40 |
| 2075 | T | C | 692 | V | A | E | 407 | T2075C | V407A | 0.69 | 43.78 |
| 2179 | T | C | 727 | F | L | E | 442 | T2179C | F442L | 0.12 | 1.95 |
| 2238 | C | T | 746 | L | * | E | 461 | C2238T | L461* | 0.69 | 44.42 |
| 2363 | A | G | 788 | K | R | NS1 | 10 | A2363G | K10R | 0.68 | 42.88 |
| 2387 | G | A | 796 | G | D | NS1 | 18 | G2387A | G18D | 0.07 | 1.28 |
| 2497 | T | C | 833 | C | R | NS1 | 55 | T2497C | C55R | 0.11 | 2.50 |
| 2522 | T | C | 841 | L | P | NS1 | 63 | T2522C | L63P | 0.10 | 2.85 |
| 2666 | T | C | 889 | L | P | NS1 | 111 | T2666C | L111P | 0.10 | 2.28 |
| 2684 | C | T | 895 | T | I | NS1 | 117 | C2684T | T117I | 0.69 | 44.36 |
| 2813 | T | C | 938 | M | T | NS1 | 160 | T2813C | M160T | 0.69 | 49.75 |
| 3065 | C | T | 1022 | P | L | NS1 | 244 | C3065T | P244L | 0.69 | 43.48 |
| 3156 | A | G | 1052 | E | * | NS1 | 274 | A3156G | E274* | 0.68 | 41.99 |
| 3535 | C | T | 1179 | L | F | NS2A | 49 | C3535T | L49F | 0.09 | 1.82 |
| 3699 | A | G | 1233 | V | * | NS2A | 103 | A3699G | V103* | 0.48 | 19.70 |
| 3799 | A | G | 1267 | N | D | NS2A | 137 | A3799G | N137D | 0.15 | 3.56 |
| 3807 | A | T | 1269 | V | * | NS2A | 139 | A3807T | V139* | 0.44 | 17.18 |
| 3840 | C | T | 1280 | P | * | NS2A | 150 | C3840T | P150* | 0.07 | 1.69 |
| 3963 | T | C | 1321 | T | * | NS2A | 191 | T3963C | T191* | 0.24 | 5.34 |
| 4051 | T | C | 1351 | F | L | NS2A | 221 | T4051C | F221L | 0.23 | 4.78 |
| 4473 | T | C | 1491 | D | * | NS3 | 7 | T4473C | D7* | 0.31 | 9.39 |
| 4746 | A | G | 1582 | Q | * | NS3 | 98 | A4746G | Q98* | 0.45 | 16.29 |
| 4855 | A | G | 1619 | S | G | NS3 | 135 | A4855G | S135G | 0.06 | 1.07 |
| 4922 | T | C | 1641 | I | T | NS3 | 157 | T4922C | I157T | 0.09 | 2.09 |
| 4941 | C | T | 1647 | S | * | NS3 | 163 | C4941T | S163* | 0.21 | 5.98 |
| 5035 | A | G | 1679 | I | V | NS3 | 195 | A5035G | I195V | 0.07 | 1.96 |
| 5422 | C | T | 1808 | P | S | NS3 | 324 | C5422T | P324S | 0.33 | 8.12 |
| 5524 | G | A | 1842 | A | T | NS3 | 358 | G5524A | A358T | 0.33 | 10.98 |
| 5571 | A | G | 1857 | A | * | NS3 | 373 | A5571G | A373* | 0.18 | 5.26 |
| 5608 | A | G | 1870 | S | G | NS3 | 386 | A5608G | S386G | 0.13 | 3.89 |
| 5632 | A | G | 1878 | T | A | NS3 | 394 | A5632G | T394A | 0.14 | 2.91 |
| 5776 | G | T | 1926 | G | W | NS3 | 442 | G5776T | G442W | 0.24 | 6.62 |
| 5802 | A | G | 1934 | P | * | NS3 | 450 | A5802G | P450* | 0.19 | 5.53 |
| 6025 | G | A | 2009 | G | S | NS3 | 525 | G6025A | G525S | 0.09 | 1.40 |
| 6208 | T | C | 2070 | C | R | NS3 | 586 | T6208C | C586R | 0.06 | 1.08 |
| 6214 | G | A | 2072 | A | T | NS3 | 588 | G6214A | A588T | 0.21 | 4.80 |
| 6286 | C | T | 2096 | L | * | NS3 | 612 | C6286T | L612* | 0.37 | 11.12 |
| 6621 | A | G | 2107 | R | * | NS4A | 1 | A6621G | R1* | 0.06 | 1.67 |
| 6861 | T | C | 2287 | D | * | NS4B | 31 | T6861C | D31* | 0.27 | 6.82 |
| 6929 | T | C | 2310 | L | S | NS4B | 54 | T6929C | L54S | 0.21 | 5.66 |
| 6942 | C | T | 2314 | I | * | NS4B | 58 | C6942T | I58* | 0.19 | 5.41 |
| 7049 | T | C | 2350 | I | T | NS4B | 94 | T7049C | I94T | 0.18 | 5.15 |
| 7061 | T | C | 2354 | V | A | NS4B | 98 | T7061C | V98A | 0.08 | 1.20 |
| 7157 | A | G | 2386 | Q | R | NS4B | 130 | A7157G | Q130R | 0.08 | 1.83 |
| 7207 | C | T | 2403 | P | S | NS4B | 147 | C7207T | P147S | 0.46 | 17.02 |
| 7347 | G | A | 2449 | L | * | NS4B | 193 | G7347A | L193* | 0.36 | 12.99 |
| 7524 | T | C | 2508 | S | * | NS5 | 2 | T7524C | S2* | 0.36 | 12.32 |
| 7527 | G | A | 2509 | A | * | NS5 | 3 | G7527A | A3* | 0.53 | 25.78 |
| 7580 | A | G | 2527 | K | R | NS5 | 21 | A7580G | K21R | 0.07 | 2.19 |
| 7974 | G | A | 2658 | S | * | NS5 | 152 | G7974A | S152* | 0.45 | 15.35 |
| 8275 | C | T | 2759 | L | * | NS5 | 253 | C8275T | L253* | 0.33 | 9.18 |
| 8283 | T | C | 2761 | A | * | NS5 | 255 | T8283C | A255* | 0.06 | 1.06 |
| 8460 | C | T | 2820 | T | * | NS5 | 314 | C8460T | T314* | 0.39 | 12.01 |
| 8510 | A | G | 2837 | Y | C | NS5 | 331 | A8510G | Y331C | 0.33 | 10.16 |
| 8799 | C | T | 2933 | V | * | NS5 | 427 | C8799T | V427* | 0.30 | 7.39 |
| 8812 | T | C | 2938 | F | L | NS5 | 432 | T8812C | F432L | 0.15 | 3.65 |
| 8882 | T | C | 2961 | M | T | NS5 | 455 | T8882C | M455T | 0.06 | 1.23 |
| 9143 | G | A | 3048 | R | H | NS5 | 542 | G9143A | R542H | 0.31 | 10.75 |
| 9292 | A | T | 3098 | M | L | NS5 | 592 | A9292T | M592L | 0.05 | 1.17 |
| 10232 | T | C | - | - | - | 3'UTR |  | T10232C | -- | 0.08 | 2.04 |
| 10314 | T | A | - | - | - | 3'UTR |  | T10314A | -- | 0.65 | 29.00 |
| 10682 | A | G | - | - | - | 3'UTR |  | A10682G | -- | 0.68 | 42.59 |
