# Supplementary material for: Attenuation of Live-Attenuated Yellow Fever 17D Vaccine Virus Is Localized to a High-Fidelity Replication Complex
Source: mBio. 2019 Oct 22;10(5):e02294-19. doi: 10.1128/mBio.02294-19 (PMC6805994; doi:10.1128/mBio.02294-19)
Supplement: TABLE S2 [file mBio.02294-19-st002.docx]

**Supplementary table 2: FNV treated with ribavirin generates less SNV than ribavirin FVV.** Variants were detected using VPhaserII with variants below 1% frequency and those that did not pass the strand bias test were discarded. Variants highlighted in green are common throughout samples.

| FNV 0 uM |  |  |  |  |  |  |  |  |  |  |  |
| --- | --- | --- | --- | --- | --- | --- | --- | --- | --- | --- | --- |
| CDS Position | Consensus | Variant | Protein Position | Consensus | Variant | Gene | Codon within Gene | Nucleotide | Amino Acid | Mutational Freq | SNV Percentage |
| 436 | A | G | 146 | M | V | M | 25 | A436G | M25V | 0.11 | 2.35 |
| 1452 | A | G | 484 | E | * | E | 199 | A1452G | E199* | 0.14 | 3.21 |
| 1983 | T | C | 661 | I | * | E | 376 | T1983C | I376* | 0.11 | 2.30 |
| 2050 | T | C | 684 | L | * | E | 399 | T2050C | L399* | 0.01 | 8.61 |
| 2492 | G | A | 831 | G | E | NS1 | 53 | G2492A | G53E | 0.29 | 13.00 |
| 2955 | A | G | 985 | V | * | NS1 | 207 | A2955G | V207* | 0.39 | 10.14 |
| 3183 | C | T | 1061 | T | * | NS1 | 283 | C3183T | T283* | 0.35 | 14.14 |
| 3227 | C | T | 1076 | A | V | NS1 | 298 | C3227T | A298V | 0.07 | 1.34 |
| 5133 | C | T | 1711 | A | * | NS3 | 227 | C5133T | A227* | 0.51 | 20.53 |
| 5262 | A | G | 1754 | L | * | NS3 | 270 | A5262G | L270* | 0.07 | 1.27 |
| 6005 | C | T | 2002 | T | I | NS3 | 518 | C6005T | T518I | 0.06 | 1.16 |
| 6162 | C | T | 2054 | G | * | NS3 | 570 | C6162T | G570* | 0.07 | 1.36 |
| 6315 | T | C | 2105 | G | * | NS3 | 621 | T6315C | G621* | 0.14 | 3.03 |
| 6660 | C | T | 2220 | V | * | NS4A | 113 | C6660T | V113* | 0.06 | 1.17 |
| 6798 | A | G | 2266 | K | * | NS4B | 10 | A6798G | K10* | 0.30 | 8.95 |
| 7982 | T | C | 2661 | V | A | NS5 | 155 | T7982C | V155A | 0.51 | 21.06 |
| 8457 | A | G | 2819 | K | * | NS5 | 313 | A8457G | K313* | 0.08 | 1.53 |
| 8490 | T | A | 2830 | G | * | NS5 | 324 | T8490A | G324* | 0.34 | 10.30 |
| 9177 | G | A | 3059 | E | * | NS5 | 553 | G9177A | E553* | 0.65 | 35.78 |
| 9447 | C | T | 3149 | V | * | NS5 | 643 | C9447T | V643* | 0.43 | 15.20 |
| 9585 | C | T | 3159 | V | * | NS5 | 653 | C9585T | V653* | 0.15 | 3.47 |
| 9759 | A | G | 3253 | G | * | NS5 | 747 | A9759G | G747* | 0.32 | 9.21 |
| 10269 | A | G | - | - | - | 3'UTR | - | A10269G | --- | 0.30 | 9.09 |
| 10276 | G | C | - | - | - | 3'UTR | - | G10276C | --- | 0.08 | 1.50 |
| 10406 | G | C | - | - | - | 3'UTR | - | G10406C | --- | 0.12 | 2.53 |
|  |  |  |  |  |  |  |  |  |  |  |  |
| FNV 0.5 uM |  |  |  |  |  |  |  |  |  |  |  |
| CDS Position | Consensus | Variant | Protein Position | Consensus | Variant | Gene | Codon within Gene | Nucleotide | Amino Acid | Mutational Freq | SNV Percentage |
| 436 | A | G | 146 | M | V | M | 24 | A436G | M24V | 0.15 | 3.31 |
| 736 | T | C | 246 | F | L | M | 124 | T736C | F124L | 0.09 | 1.47 |
| 1452 | A | G | 484 | E | * | E | 199 | A1452G | E199* | 0.14 | 3.27 |
| 1983 | T | C | 661 | I | * | E | 376 | T1983C | I376* | 0.12 | 2.70 |
| 2050 | T | C | 684 | L | * | E | 399 | T2050C | L399* | 0.28 | 8.19 |
| 2226 | A | G | 742 | I | M | E | 457 | A2226G | I457M | 0.07 | 1.35 |
| 2466 | G | A | 822 | S | * | NS1 | 44 | G2466A | S44* | 0.06 | 1.10 |
| 2492 | G | A | 831 | G | E | NS1 | 53 | G2492A | G53E | 0.43 | 15.02 |
| 2955 | A | G | 985 | V | * | NS1 | 207 | A2955G | V207* | 0.31 | 9.48 |
| 3183 | C | T | 1061 | T | * | NS1 | 283 | C3183T | T283* | 0.41 | 14.50 |
| 5133 | C | T | 1711 | A | * | NS3 | 227 | C5133T | A227* | 0.51 | 20.43 |
| 6315 | T | C | 2105 | G | * | NS3 | 621 | T6315C | G621* | 0.12 | 2.62 |
| 6642 | A | C | 2214 | I | * | NS4A | 107 | A6642C | I107* | 0.09 | 1.44 |
| 7982 | T | C | 2661 | V | A | NS5 | 155 | T7982C | V155A | 0.50 | 20.15 |
| 8490 | T | A | 2830 | G | * | NS5 | 324 | T8490A | G324* | 0.35 | 10.07 |
| 9177 | G | A | 3059 | E | * | NS5 | 553 | G9177A | E553* | 0.65 | 34.87 |
| 9447 | C | T | 3149 | V | * | NS5 | 643 | C9447T | V643* | 0.46 | 17.23 |
| 9585 | C | T | 3159 | V | * | NS5 | 653 | C9585T | V653* | 0.13 | 2.84 |
| 9759 | A | G | 3253 | G | * | NS5 | 747 | A9759G | G747* | 0.31 | 8.42 |
| 10266 | C | T | - | - | - | 3'UTR | - | C10266T | --- | 0.06 | 1.06 |
| 10269 | A | G | - | - | - | 3'UTR | - | A10269G | --- | 0.30 | 9.04 |
| 10276 | G | C | - | - | - | 3'UTR | - | G10276C | --- | 0.07 | 1.28 |
| 10286 | A | G | - | - | - | 3'UTR | - | A10286G | --- | 0.07 | 1.31 |
| 10319 | C | T | - | - | - | 3'UTR | - | C10319T | --- | 0.10 | 2.10 |
|  |  |  |  |  |  |  |  |  |  |  |  |
| FVV 0 uM |  |  |  |  |  |  |  |  |  |  |  |
| CDS Position | Consensus | Variant | Protein Position | Consensus | Variant | Gene | Codon within Gene | Nucleotide | Amino Acid | Mutational Freq | SNV Percentage |
| -60 | T | C | - | - | - | 5'UTR | - | - | --- | 0.09 | 1.89 |
| 1632 | C | T | 544 | T | * | E | 259 | C1632T | T259* | 0.02 | 1.05 |
| 1847 | A | G | 616 | K | R | E | 331 | A1847G | K331R | 0.20 | 5.01 |
| 2075 | C | T | 692 | A | V | E | 407 | C2075T | A407V | 0.66 | 35.34 |
| 3938 | C | T | 1313 | S | F | NS2A | 183 | C3938T | S183F | 0.06 | 1.05 |
| 4098 | T | C | 1366 | G | * | NS2B | 12 | T4098C | G12* | 0.14 | 3.22 |
| 4251 | G | A | 1417 | E | * | NS2B | 63 | G4251A | E63* | 0.07 | 1.01 |
| 4387 | A | C | 1463 | I | L | NS2B | 109 | A4387C | I109L | 0.07 | 1.26 |
| 5562 | C | T | 1854 | I | * | NS3 | 370 | C5562T | I370* | 0.64 | 34.51 |
| 6528 | C | T | 2176 | I | * | NS4A | 69 | C6528T | I69* | 0.08 | 1.45 |
| 7504 | G | A | 2502 | E | K | NS4B | 246 | G7504A | E246K | 0.27 | 7.46 |
| 7506 | A | C | 2502 | E | D | NS4B | 246 | A7506C | E246D | 0.07 | 1.36 |
| 7523 | G | T | 2508 | R | I | NS5 | 2 | G7523T | R2I | 0.19 | 4.77 |
| 7524 | A | T | 2508 | R | S | NS5 | 2 | A7524T | R2S | 0.10 | 1.27 |
| 7583 | A | G | 2528 | Q | R | NS5 | 22 | A7583G | Q22R | 0.06 | 1.13 |
| 8181 | C | T | 2727 | Y | * | NS5 | 221 | C8181T | Y221* | 0.07 | 1.21 |
| 9022 | G | A | 3008 | E | K | NS5 | 502 | G9022A | E502K | 0.20 | 4.89 |
| 9776 | A | G | 3259 | K | R | NS5 | 753 | A9776G | K753R | 0.07 | 1.30 |
| 10024 | G | A | 3342 | E | K | NS5 | 836 | G10024A | E836K | 0.08 | 1.18 |
| 10249 | T | C | - | - | - | 3'UTR | - | T10249C | --- | 0.06 | 1.12 |
| 10271 | C | T | - | - | - | 3'UTR | - | C10271T | --- | 0.09 | 1.17 |
|  |  |  |  |  |  |  |  |  |  |  |  |
| FVV 0.5 uM |  |  |  |  |  |  |  |  |  |  |  |
| CDS Position | Consensus | Variant | Protein Position | Consensus | Variant | Gene | Codon within Gene | Nucleotide | Amino Acid | Mutational Freq | SNV Percentage |
| 174 | A | G | 58 | G | * | C | 58 | A174G | G58* | 0.44 | 15.78 |
| 186 | G | A | 62 | T | * | C | 62 | G186A | T62* | 0.67 | 40.12 |
| 252 | T | C | 84 | V | * | C | 84 | T252C | V84* | 0.68 | 42.17 |
| 736 | C | T | 246 | L | F | M | 125 | C736T | L125F | 0.65 | 34.63 |
| 765 | A | G | 255 | T | * | M | 134 | A765G | T134* | 0.68 | 43.00 |
| 1022 | C | T | 341 | A | V | E | 56 | C1022T | A56V | 0.68 | 41.03 |
| 1345 | C | T | 452 | P | S | E | 167 | C1345T | P167S | 0.09 | 1.92 |
| 1364 | C | T | 455 | A | V | E | 170 | C1364T | A170V | 0.66 | 37.57 |
| 1373 | C | T | 458 | T | I | E | 173 | C1373T | T173I | 0.68 | 39.63 |
| 1632 | C | T | 544 | T | * | E | 259 | C1632T | T259* | 0.67 | 40.22 |
| 1752 | G | A | 584 | M | I | E | 299 | G1752A | M299I | 0.66 | 36.66 |
| 1769 | C | T | 590 | S | F | E | 305 | C1769T | S305F | 0.68 | 36.34 |
| 1828 | C | T | 610 | P | S | E | 325 | C1828T | P325S | 0.68 | 42.64 |
| 1847 | A | G | 616 | K | R | E | 331 | A1847G | K331R | 0.69 | 49.83 |
| 1974 | T | C | 658 | D | * | E | 373 | T1974C | D373* | 0.69 | 41.56 |
| 1977 | C | T | 659 | S | * | E | 374 | C1977T | S374* | 0.17 | 3.97 |
| 2238 | C | T | 746 | L | * | E | 461 | C2238T | L461* | 0.69 | 45.40 |
| 2569 | C | T | 857 | L | F | NS1 | 79 | C2569T | L79F | 0.70 | 46.62 |
| 3253 | A | G | 1085 | I | V | NS1 | 307 | A3253G | I307V | 0.69 | 40.92 |
| 3742 | A | G | 1248 | M | V | NS2A | 118 | A3742G | M118V | 0.68 | 42.47 |
| 3889 | A | G | 1297 | T | A | NS2A | 167 | A3889G | T167A | 0.69 | 48.16 |
| 3895 | C | T | 1299 | L | F | NS2A | 169 | C3895T | L169F | 0.69 | 46.72 |
| 3904 | A | G | 1302 | T | A | NS2A | 172 | A3904G | T172A | 0.68 | 41.62 |
| 3936 | C | T | 1312 | N | * | NS2A | 182 | C3936T | N182* | 0.67 | 40.11 |
| 3938 | C | T | 1313 | S | F | NS2A | 183 | C3938T | S183F | 0.68 | 40.77 |
| 4098 | T | C | 1366 | G | * | NS2B | 12 | T4098C | G12* | 0.12 | 2.62 |
| 4171 | A | C | 1391 | I | L | NS2B | 37 | A4171C | I37L | 0.68 | 41.01 |
| 4269 | A | G | 1423 | G | * | NS2B | 69 | A4269G | G69* | 0.68 | 41.18 |
| 4494 | T | C | 1498 | I | * | NS3 | 14 | T4494C | I14* | 0.65 | 36.16 |
| 4590 | G | A | 1530 | G | * | NS3 | 46 | G4590A | G46* | 0.13 | 2.94 |
| 4755 | T | G | 1585 | A | * | NS3 | 101 | T4755G | A101* | 0.65 | 35.56 |
| 5035 | A | G | 1679 | I | V | NS3 | 195 | A5035G | I195V | 0.68 | 41.73 |
| 5076 | T | C | 1692 | F | * | NS3 | 208 | T5076C | F208* | 0.66 | 38.07 |
| 5244 | C | T | 1748 | A | * | NS3 | 264 | C5244T | A264* | 0.69 | 44.53 |
| 5290 | A | G | 1764 | I | V | NS3 | 280 | A5290G | I280V | 0.69 | 43.75 |
| 5313 | C | T | 1771 | I | * | NS3 | 287 | C5313T | I287* | 0.67 | 37.61 |
| 5355 | C | T | 1785 | A | * | NS3 | 301 | C5355T | A301* | 0.68 | 42.50 |
| 5808 | C | T | 1936 | R | * | NS3 | 452 | C5808T | R452* | 0.70 | 45.69 |
| 5905 | G | A | 1969 | D | N | NS3 | 485 | G5905A | D485N | 0.68 | 41.29 |
| 6330 | G | T | 2110 | A | * | NS4A | 3 | G6330T | A3* | 0.68 | 40.84 |
| 6758 | T | C | 2253 | V | A | NS4A | 146 | T6758C | V146A | 0.68 | 43.47 |
| 7107 | A | G | 2370 | G | R | NS4B | 114 | A7107G | G114R | 0.12 | 2.64 |
| 7378 | T | C | 2460 | L | * | NS4B | 204 | T7378C | L204* | 0.69 | 42.12 |
| 7453 | C | A | 2485 | R | * | NS4B | 229 | C7453A | R229* | 0.69 | 41.74 |
| 7462 | T | C | 2488 | Y | H | NS4B | 232 | T7462C | Y232H | 0.68 | 42.73 |
| 7504 | G | A | 2502 | E | K | NS4B | 246 | G7504A | E246K | 0.69 | 47.34 |
| 7523 | G | T | 2508 | R | I | NS5 | 2 | G7523T | R2I | 0.13 | 2.75 |
| 7524 | A | C | 2508 | R | S | NS5 | 2 | A7524C | R2S | 0.70 | 42.32 |
| 7583 | A | G | 2528 | Q | R | NS5 | 22 | A7583G | Q22R | 0.67 | 38.71 |
| 7827 | C | T | 2609 | F | * | NS5 | 103 | C7827T | F103* | 0.68 | 38.57 |
| 7890 | T | C | 2630 | I | * | NS5 | 124 | T7890C | I124* | 0.68 | 41.39 |
| 8505 | A | G | 2835 | L | * | NS5 | 329 | A8505G | L329* | 0.68 | 41.31 |
| 8511 | C | T | 2837 | Y | * | NS5 | 331 | C8511T | Y331* | 0.68 | 40.53 |
| 8655 | C | T | 2885 | N | * | NS5 | 379 | C8655T | N379* | 0.08 | 1.62 |
| 8904 | G | A | 2968 | K | * | NS5 | 462 | G8904A | K462* | 0.07 | 1.41 |
| 9022 | G | A | 3008 | E | K | NS5 | 502 | G9022A | E502K | 0.07 | 1.41 |
| 9852 | G | A | 3284 | L | * | NS5 | 778 | G9852A | L778* | 0.68 | 41.04 |
| 10024 | G | A | 3342 | E | K | NS5 | 836 | G10024A | E836K | 0.67 | 38.54 |
| 10167 | T | C | 3389 | Y | * | NS5 | 883 | T10167C | Y883* | 0.63 | 32.27 |
| 10194 | A | G | 3398 | R | * | NS5 | 892 | A10194G | R892* | 0.67 | 40.35 |
| 10220 | C | T | 3407 | P | L | NS5 | 901 | C10220T | P901L | 0.68 | 43.55 |
| 10249 | T | C | - | - | - | 3'UTR | - | T10249C | --- | 0.68 | 40.72 |
| 10276 | G | C | - | - | - | 3'UTR | - | G10276C | --- | 0.09 | 1.39 |
| 10300 | T | C | - | - | - | 3'UTR | - | T10300C | --- | 0.68 | 42.12 |
| 10432 | T | C | - | - | - | 3'UTR | - | T10432C | --- | 0.65 | 34.93 |
| 10544 | C | T | - | - | - | 3'UTR | - | C10544T | --- | 0.14 | 3.14 |
